# MH in Russia: obstacle races

**DOI:** 10.1186/1471-2253-14-S1-A24

**Published:** 2014-08-18

**Authors:** Angelina A Kazantseva, Konstantin M Lebedinskii

**Affiliations:** 1 Vladimir L. Vanevskii Department of Anaesthesiology and Reanimatology, Ilya I. Mechinkov North-Western State Medical University,Saint-Petersburg, 193015, Russia

## Background

The problem of malignant hyperthermia (MH) was awaken in Russia in 2012, when 3 y.o. girl died in private hospital in Saint Petersburg. MH is well known, but well forgotten by most of anaesthesiologists in Russia since we have a lack of diagnostic and treatment tools. Even if MH crisis takes place we have no specific treatment at the moment and a few opportunities for diagnosing MH susceptibility (MHS): dantrolene is still not registered, capnography isn’t available in probably 2/3 of operating theatres all over the country, there is only one genetic lab performing search for causative MH mutations and in vitro contracture test lab is not equipped with proper devices.

MH is not a part of any national governmental or healthcare ministry program, so our work is not sponsored and is totally volunteered. Our small team is full of enthusiasm, but this can’t cover expenses for lab equipment. We had received financial support from two commercial sponsors, but it was not too big and we were not able to buy all the necessary things. We had received a great support from our French colleagues who shared their experience, knowledge, soft- and hardware and even some important lab pieces.

This report is intended to be a kind of «Call for help». If your lab has some equipment to share (old, not in use and even out of service) we would appreciate any contribution and support.

